# Implementing PRISMA-P: recommendations for prospective authors

**DOI:** 10.1186/s13643-016-0191-y

**Published:** 2016-01-28

**Authors:** David Moher, Lesley Stewart, Paul Shekelle

**Affiliations:** Clinical Epidemiology Program, Ottawa Hospital Research Institute, School of Epidemiology, Public Health and Preventive Medicine, University of Ottawa, 501 Smyth Rd, Room L1288, Ottawa, ON K1H 8L6 Canada; Canadian EQUATOR Centre, Ottawa, Canada and The Ottawa Hospital, General Campus, 501 Smyth Rd, Room L1288, Ottawa, ON K1H 8L6 Canada; Centre for Reviews and Dissemination, University of York, York, YO10 5DD UK; Division of General Internal Medicine, West Los Angeles VA Medical Center, Los Angeles, California 90066 USA

Systematic reviews have become very popular. A recent estimate suggests that 22 new systematic reviews are published daily [1]. One reason for this interest is that they serve many purposes. For example, the influential Institute of Medicine has indicated that a systematic review is an essential component when developing clinical practice guidelines within the USA [2]. Some granting agencies are now advocating for the use of systematic reviews as an evidence-based rationale for the conduct of a proposed randomized trial [3]. And journals are now demanding the use of systematic reviews to provide readers with context of the results of a clinical trial [4].

For systematic reviews to be useful, they need to be reported in the highest possible quality thus facilitating their accurate use across a wide spectrum of stakeholders, including patients. Unfortunately, surveys of the published literature indicate that the quality of reporting is not optimal. For example, there is evidence indicating that reporting biases, particularly selective outcome reporting, is prevalent. An early example of differences between outcomes reported in protocols and the paired completed review was an examination of 47 Cochrane reviews in which 43 (91 %) contained a major change, such as the addition or deletion of outcomes, between the protocol and the full publication [5]. More recently, in an examination of 485 Cochrane protocol-review pairs, 38 % (95 % CI 23 to 54 %) were found to have discrepant outcomes (i.e., added, omitted, or changed the priority) between the protocol and completed review [6]. The vast majority of these discrepancies were without attribution with more significant outcomes being upgraded or added. Whether or not, and to what extent, these examples reflect reporting biases is not clear. However, they represent inconsistencies that should be avoided by authors.

The gold standard for identifying reporting biases is a comparison of the completed review with its paired protocol. Such an examination is difficult with systematic reviews as too few of them report working from a protocol, although a growing number of funders are now requiring them. Perhaps, systematic reviewers do not report or use protocols because there has been little guidance on how to report them until recently. To help facilitate the use of reporting systematic review protocols, the three of us and several colleagues developed Preferred Reporting Items for Systematic Review and Meta-Analysis Protocols (PRISMA-P) [7]. This is a reporting guideline consisting of a 17-item checklist, to help prospective authors in the preparation and reporting of a scientifically rigorous systematic review protocol. We also prepared a pedagogical explanation and elaboration document to facilitate its use [8]. Readers appear interested in the guidance. Since its publication a little more than a year ago, it has been downloaded about 45,000 times and cited (Google scholar) nearly 100 times. This journal and others have endorsed PRISMA-P. Here, we describe how the journal intends to implement it.

All protocol submissions to the journal should use continuous line numbering in their manuscript. Authors should also include a completed PRISMA-P checklist indicating whether or not the requested item information is reported (by completing the check mark). If the item is checked, authors should then specify the line number (or range of line numbers) where this information is described. Manuscripts accepted for publication will have the completed PRIMSA-P checklist (on submission) included as an Appendix to their publication, which must be referenced within the main text (Additional file 1). Prospective authors can download a Word version of the PRISMA-P checklist, which includes the two added columns, from the journal’s website (URL to be added)or the PRISMA website (ttp://www.prisma-statement.org/Extensions/Protocols.aspx). If PRISMA-P was used to help report the protocol, it should be cited or the PRISMA-P URL (http://www.prisma-statement.org/Extensions/Protocols.aspx) on the PRISMA website should be reported.

About half of what the journal publishes are protocols of systematic reviews. We want to ensure they are published to the highest possible quality. Endorsement and implementation of reporting guidelines appears to improve the completeness of reporting. For example, a systematic review examining the completeness of reporting in more than 16,000 randomized trials in journals that endorsed the CONSORT statement, compared to journals that did not, found more complete reporting [9]. Similarly, examining 300 systematic reviews published in February 2014 found that mention of PRISMA was associated with better reporting [1].

There is always a tension between an optimal implementation strategy and ensuring minimal barriers to submission for prospective authors. We believe the journal has achieved a good balance with this strategy. Protocols submitted for publication consideration from now on that do not include a completed checklist, including the two aforementioned columns, will be returned to the authors with instructions about the journal’s systematic review protocol implementation strategy and invited to resubmit their continuously line numbered manuscript and appropriately completed checklist.

## Additional file

Additional file 1:
**PRISMA-P 2015 Checklist.** This checklist has been adapted for use with protocol submissions to Systematic Reviews from Table 3 in Moher D et al: Preferred reporting items for systematic review and meta-analysis protocols (PRISMA-P) 2015 statement. Systematic Reviews 2015 4:1.
